# Visualization of Synthetic Vascular Smooth Muscle Cells in Atherosclerotic Carotid Rat Arteries by F-18 FDG PET

**DOI:** 10.1038/s41598-017-07073-3

**Published:** 2017-08-01

**Authors:** Kisoo Pahk, Chanmin Joung, Se-Mi Jung, Hwa Young Song, Ji Yong Park, Jung Woo Byun, Yun-Sang Lee, Jin Chul Paeng, Chunsook Kim, Sungeun Kim, Won-Ki Kim

**Affiliations:** 1 Department of Neuroscience, Korea University College of Medicine, Seoul, Korea; 2 Department of Nuclear Medicine, Korea University Anam Hospital, Seoul, Korea; 3 Department of Nuclear Medicine, Seoul National University College of Medicine, Seoul, Korea; 4 Department of Biomedical Sciences, Seoul National University Graduate School, Seoul, Korea; 5 Department of Molecular Medicine and Biopharmaceutical Sciences, Graduate School of Convergence Science and Technology, and College of Medicine, Seoul National University, Seoul, Korea; 6 Department of Nuclear Medicine, Seoul National University Hospital, Seoul, Korea; 7 Department of Nursing, Kyungdong University, Wonju, Korea

## Abstract

Synthetic vascular smooth muscle cells (VSMCs) play important roles in atherosclerosis, in-stent restenosis, and transplant vasculopathy. We investigated the synthetic activity of VSMCs in the atherosclerotic carotid artery using ^18^F-fluorodeoxyglucose (FDG) positron emission tomography (PET). Atherosclerosis was induced in rats by partial ligation of the right carotid artery coupled with an atherogenic diet and vitamin D injections (2 consecutive days, 600,000 IU/day). One month later, rats were imaged by F-18 FDG PET. The atherosclerotic right carotid arteries showed prominent luminal narrowing with neointimal hyperplasia. The regions with neointimal hyperplasia were composed of α-smooth muscle actin-positive cells with decreased expression of smooth muscle myosin heavy chain. Surrogate markers of synthetic VSMCs such as collagen type III, cyclophilin A, and matrix metallopeptidase-9 were increased in neointima region. However, neither macrophages nor neutrophils were observed in regions with neointimal hyperplasia. F-18 FDG PET imaging and autoradiography showed elevated FDG uptake into the atherosclerotic carotid artery. The inner vessel layer showed higher tracer uptake than the outer layer. Consistently, the expression of glucose transporter 1 was highly increased in neointima. The present results indicate that F-18 FDG PET may be a useful tool for evaluating synthetic activities of VSMCs in vascular remodeling disorders.

## Introduction

Vascular smooth muscle cells (VSMCs) play important roles in the pathophysiological processes of various vascular disorders, such as atherosclerosis, in-stent restenosis, and transplant vasculopathy^1^. Based on their functions such as contraction, migration, or pro-inflammation, VSMCs can be subdivided into contractile or synthetic phenotypes^2, 3^. In the normal state, the majority of VSMCs in blood vessels exhibit the contractile phenotype as opposed to the synthetic phenotype^2, 3^. However, in vascular injury or in the inflammatory state, VSMCs switch from the contractile to the synthetic phenotype^2, 3^. Synthetic VSMCs migrate to the intima and form neointimal hyperplasia with inflammatory characteristics^2, 3^. Thus, targeting synthetic VSMCs is an attractive therapeutic strategy for treating vascular remodeling disorders^1^.

Several molecular imaging studies have reported significant increases of VSMC and macrophage populations in neointimal regions in patients with atherosclerosis^4, 5^ and in a rabbit model of atherosclerosis^6^. However, none of these studies examined VSMC phenotype. Pyla *et al*.^7^ reported that synthetic VSMCs exhibit high expression of facilitative glucose transporters (GLUTs), including GLUT1, a well-known FDG transporter^8^. Thus, measurement of glucose uptake may be a useful tool for distinguishing synthetic from contractile VSMCs.

Partial carotid artery ligation is a well-established technique for generating an animal model of atherosclerosis^9^. Wei *et al*.^10^ reported that the neointima in rats subjected to partial carotid artery ligation exhibited a high population of VSMCs. ^18^F-fluorodeoxyglucose (FDG) positron emission tomography (PET) is an established noninvasive image modality for measuring vascular glucose consumption^4^. In the present study, therefore, we investigated synthetic VSMC activities using F-18 FDG PET in an atherosclerotic rat model of partial carotid artery ligation.

## Results

### Histopathology

**Figure 1:**
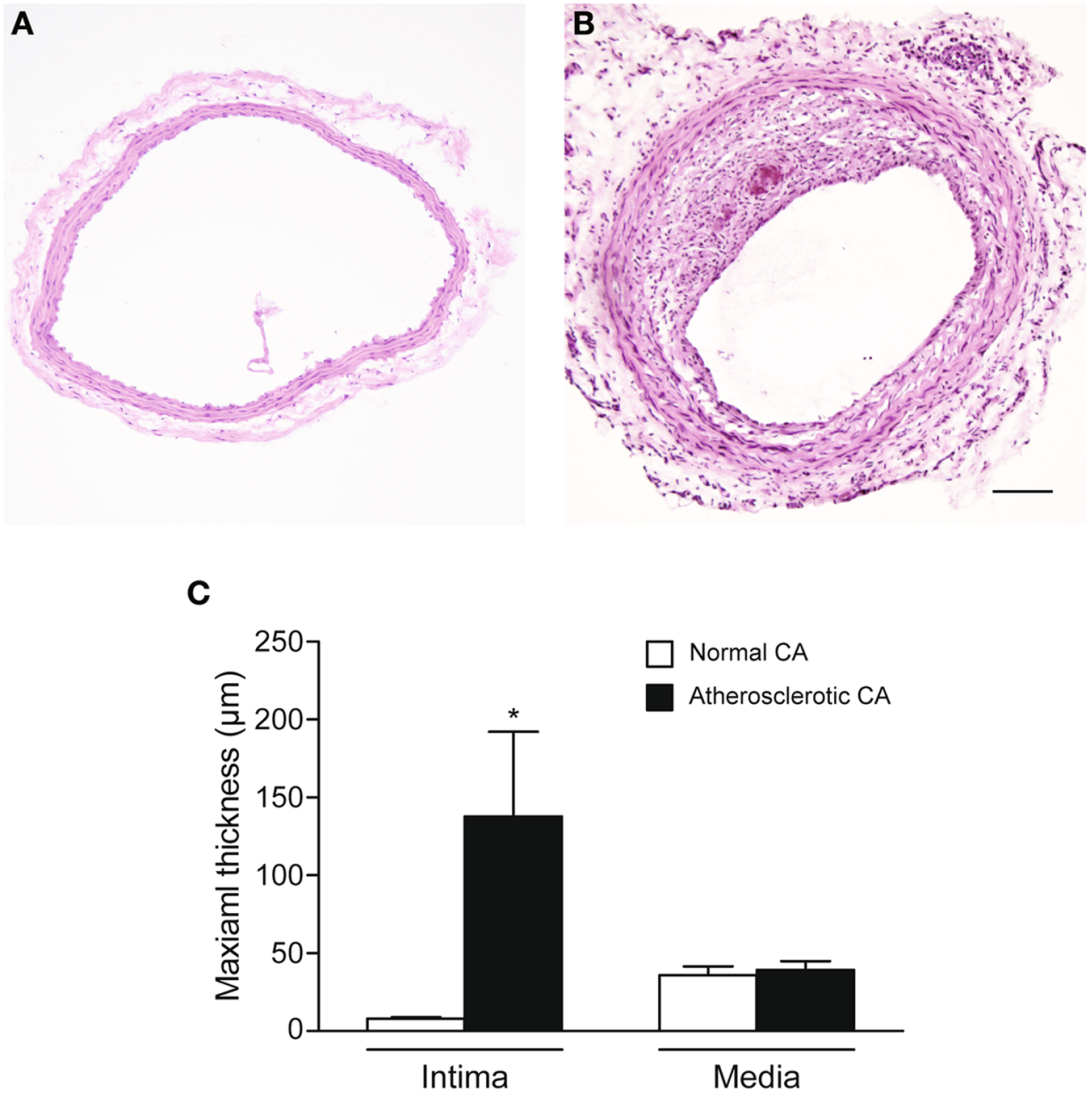
Hematoxylin and eosin (H&E) staining of harvested carotid artery (CA). (**A**) Normal CA. (**B**) Atherosclerotic CA. (**C**) Comparison of maximal wall thickness in atherosclerotic and normal CA. *p < 0.05 vs. normal control (n = 4 for each group). Scale bar, 100 µm. Magnification, ×100.

The atherosclerotic right carotid arteries presented prominent luminal narrowing with neointimal hyperplasia, while the normal carotid arteries showed no neointimal hyperplasia (Fig. 1A and B). The maximal intimal thickness of atherosclerotic arteries and normal arteries were 137.72 ± 54.46 (mean ± SD; standard deviation) µm and 8.04 ± 0.87 (mean ± SD) µm, respectively (*p* = 0.021, Fig. 1C). The maximal media thickness of atherosclerotic arteries and normal arteries were 39.2 ± 5.63 (mean ± SD) µm and 35.86 ± 5.74 (mean ± SD) µm, respectively (*p* = 0.49, Fig. 1C).

**Figure 2:**
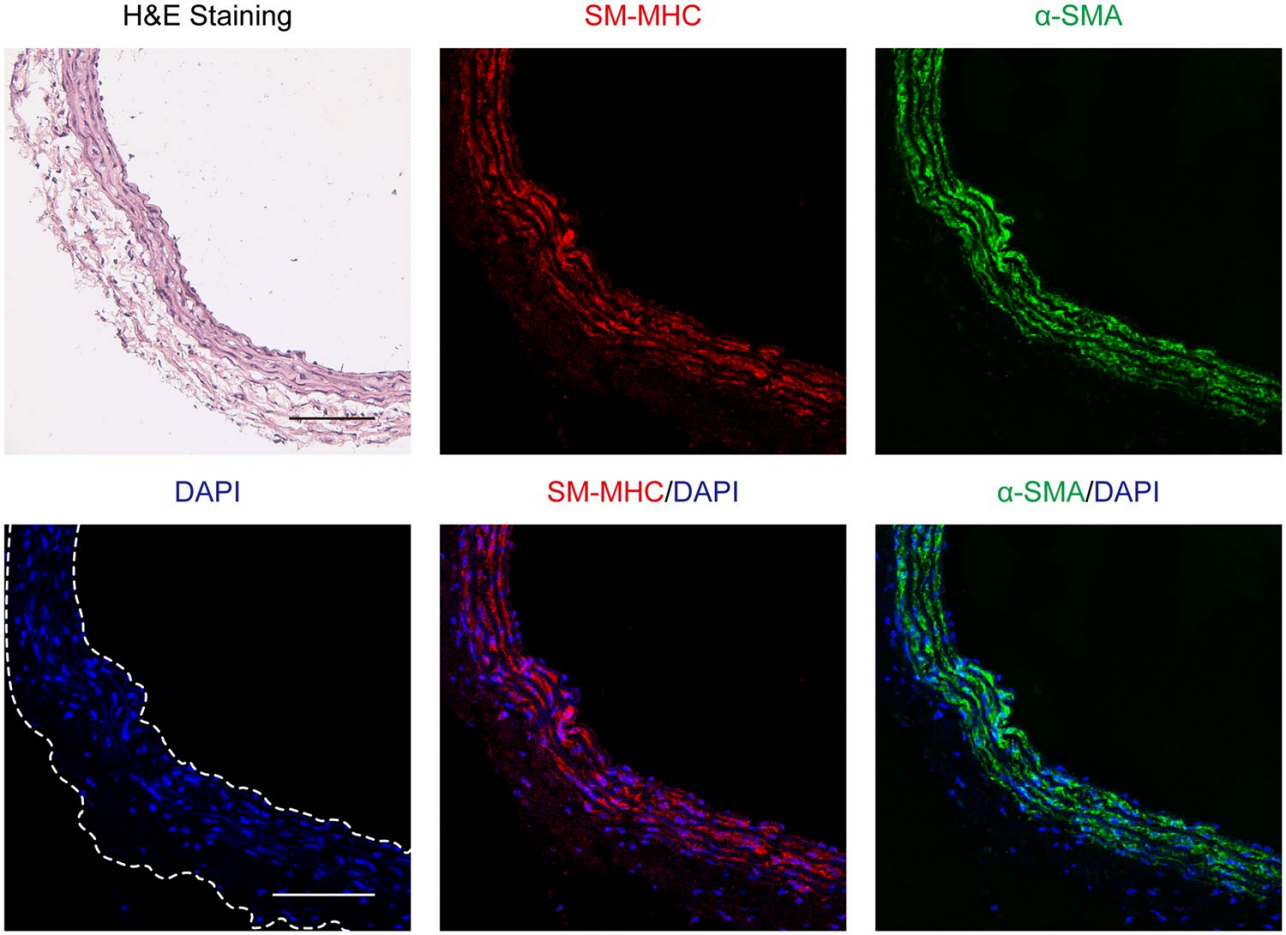
Characterization of harvested normal artery (n = 4). Images of arteries immunostained with antibodies against smooth muscle myosin heavy chain (SM-MHC) or α-smooth muscle actin (α-SMA). Nuclei were stained with DAPI. Scale bars, 100 µm. Magnification, ×100.

**Figure 3:**
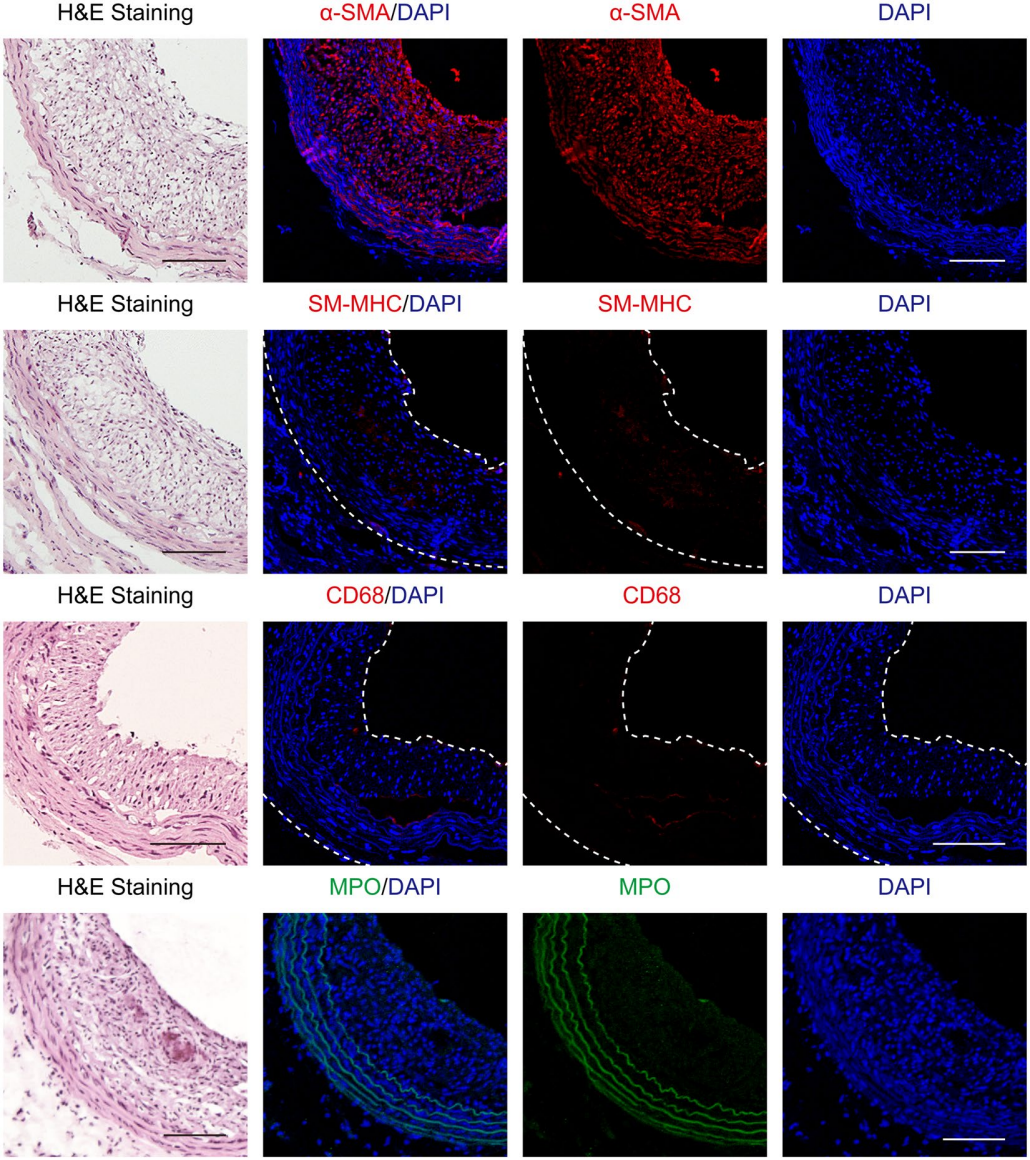
Characterization of harvested atherosclerotic right carotid arteries (n = 4). Images of arteries immunostained with antibodies against α-SMA, SM-MHC, CD68, or myeloperoxidase (MPO). Nuclei were stained with DAPI. Scale bars, 100 µm. Magnification, ×100.

**Figure 4:**
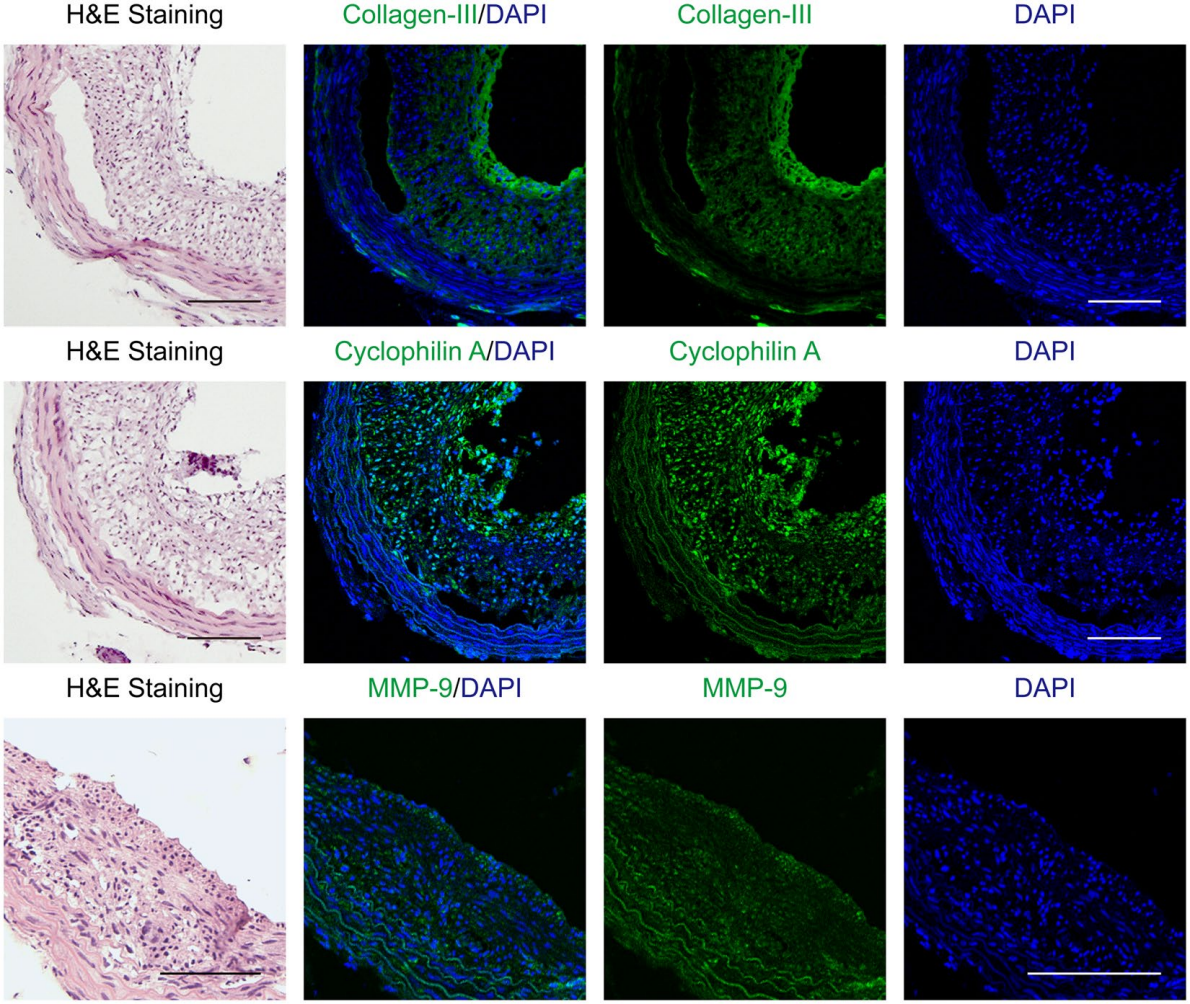
Surrogate markers of synthetic vascular smooth muscle cells in atherosclerotic right carotid arteries (n = 4). Images of arteries immunostained with antibodies against collagen type III, cyclophilin A or matrix metallopeptidase-9 (MMP-9). Nuclei were stained with DAPI. Scale bars, 100 µm. Magnification, ×100.

Normal artery was composed of smooth muscle myosin heavy chain (SM-MHC) and α-smooth muscle actin (α-SMA)-positive VSMCs in media (Fig. 2). The neointima in atherosclerotic right carotid artery exhibited many α-SMA-positive VSMCs, whereas SM-MHC-positive VSMCs, CD68-positive macrophages or myeloperoxidase (MPO)-positive neutrophils were scantly observed (Fig. 3). The neointimas exhibited significant collagen type III deposition (Fig. 4), which has been shown to be mainly excreted by synthetic VSMCs^11^. Furthermore, the thickened neointima showed increased expression of cyclophilin A and matrix metallopeptidase-9 (MMP-9) (Fig. 4), both of which are established hallmarks of synthetic VSMC activity^12, 13^.

### F-18 FDG PET

**Figure 5:**
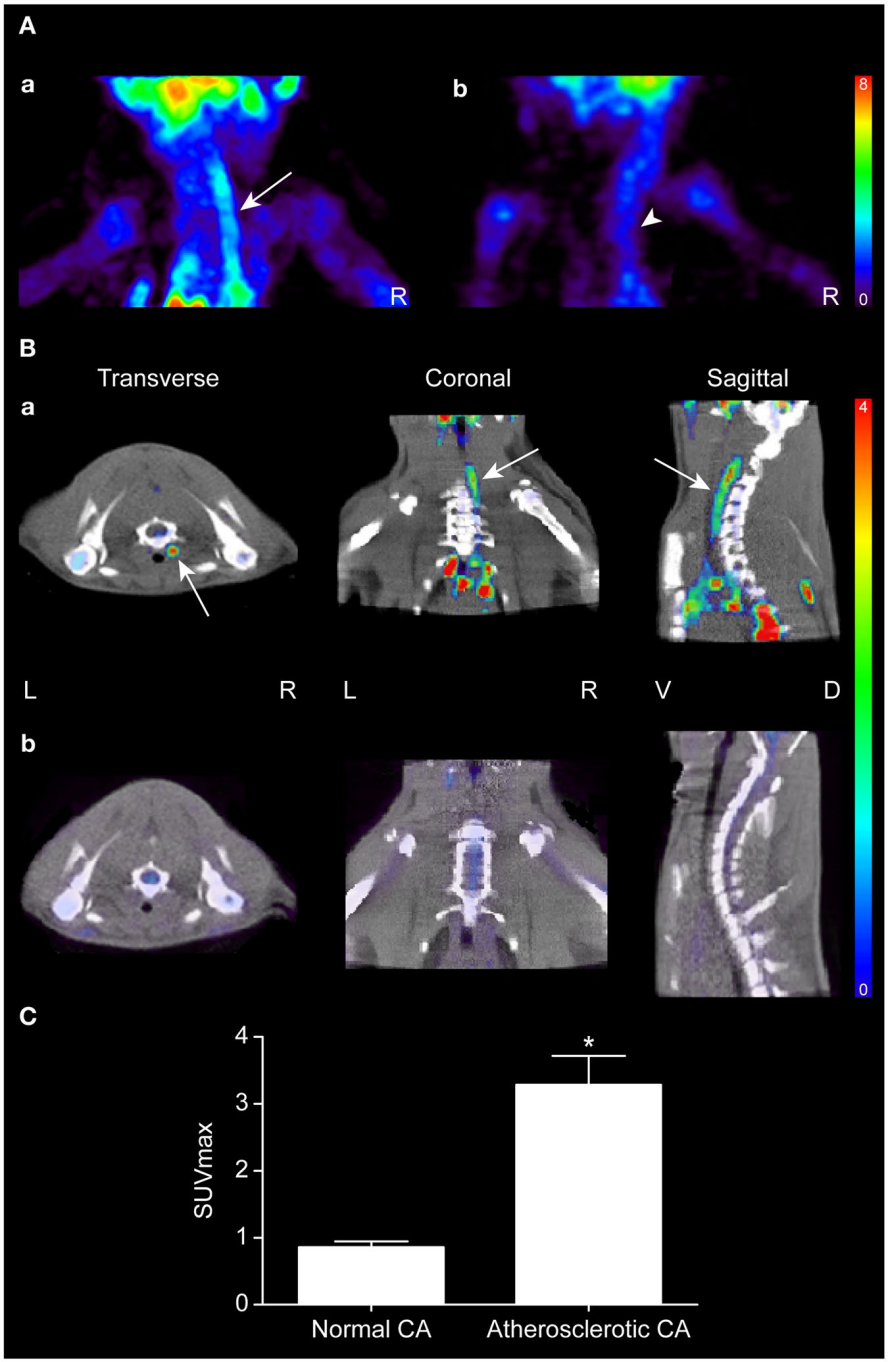
F-18 FDG PET images. (**A**) Maximum intensity projection (MIP) views of (a) an atherosclerotic rat and (b) a normal rat. (**B**) Three-plane views of (a) an atherosclerotic rat and (b) a normal rat. The arrow indicates FDG uptake of the right carotid artery and the arrow head indicates FDG uptake of the spinal bone marrow. R; right, L; left, V; ventral, D; dorsal. (**C**) Comparison of maximal standardized uptake values (SUVmax) in atherosclerotic and normal carotid arteries (CAs). *p < 0.05 vs. normal control (n = 4 for each group).

Increased FDG uptake was observed along the atherosclerotic (right) carotid artery (Fig. 5A and B). The corresponding maximum SUV was 3.28 ± 0.43 (mean ± SD). As shown in the transverse view in Fig. 5B-a, the inner circular layer of the right carotid artery showed higher tracer uptake than the outer layer. In the normal control group, no FDG uptake was apparent in either the right or left carotid artery (Fig. 5A and B). The corresponding maximum SUV was 0.86 ± 0.1 (mean ± SD). Atherosclerotic carotid artery showed significantly higher maximum SUV than the normal control group (*p* = 0.029, Fig. 5C).

### Autoradiography and glucose transporter 1 (GLUT1)

**Figure 6:**
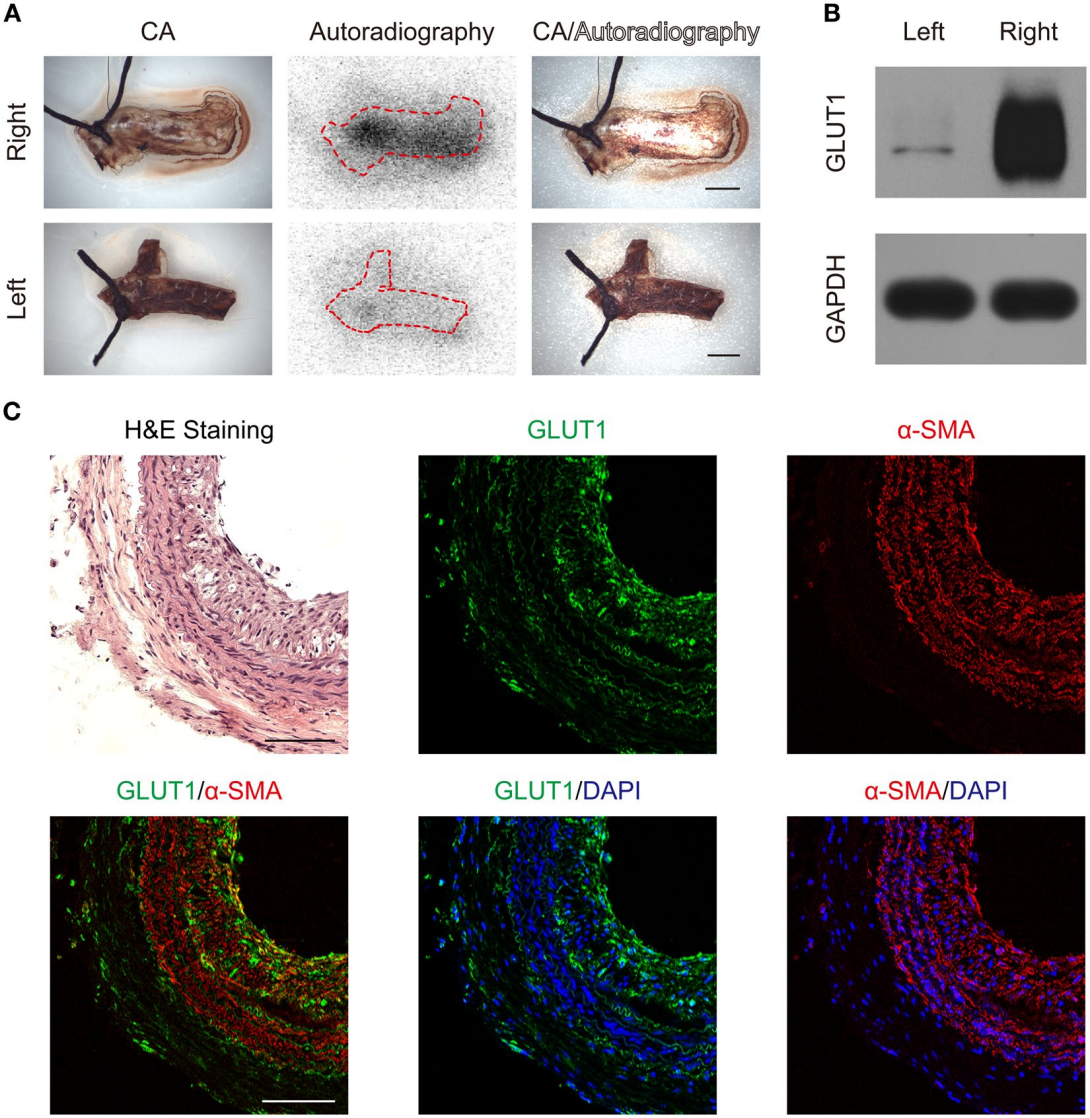
(**A**) Autoradiography scans of harvested carotid arteries (CAs). In merged images (represented as CA/Autoradiography), radioactivity signals were inversely adapted for better visualization. Scale bars, 1 mm. (**B**) Increased GLUT1 expression in atherosclerotic right carotid artery. Full-length blots are presented in Supplementary Figure 1. (**C**) Increased GLUT1 expression in synthetic VSMCs in neointima region of atherosclerotic right carotid artery. Nuclei were stained with DAPI. Scale bars, 100 µm. Magnification, ×100.

Consistent with the *in vivo* PET imaging, autoradiography showed much higher radioactivity in the right carotid artery than in the left carotid artery (Fig. 6A). Furthermore, higher radioactivity was observed in the inner layer compared to the outer layer. Western blotting showed increased GLUT1 expression in atherosclerotic right carotid artery (Fig. 6B). Immunohistochemistry further demonstrated that GLUT1 expression was increased by synthetic VSMCs in neointimal region of atherosclerotic right carotid artery (Fig. 6C).

## Discussion

In the atherosclerotic rat model used in the present study, the neointima became hypertrophic and exhibited a large VSMC population. However, few inflammatory cells such as macrophages or neutrophils were observed. Our findings are similar to those previously found in a high fat diet-induced atherosclerotic rat model^10^. Using F-18 FDG PET, we found that VSMCs from the neointima exhibited the synthetic phenotype rather than the contractile phenotype.

When blood vessels are damaged, VSMCs can switch from the contractile to the synthetic phenotype^2, 3^. During the process of neointimal hyperplasia, SM-MHC which is a well-known marker of contractile VSMC is decreased in neointima whereas α-SMA expression is preserved ^14–16^. Our results are consistent with these previous studies. In the present study, we further found that surrogate markers of synthetic VSMCs such as collagen type III, cyclophilin A, and MMP-9 were increased in the neointima. Therefore, synthetic VSMCs appear to constitute the majority of the neonitmal hyperplasia region.

In general, glucose uptake is retained in normal VSMCs^17^ and is increased by inflammatory stimuli^18^. As expected, F-18 FDG PET imaging and autoradiography revealed prominent uptake of FDG in the right carotid artery of atherosclerotic rats. However, FDG uptake was not observed in the right carotid artery of normal rats. Interestingly, FDG distribution appeared to be stratified in the atherosclerotic right carotid artery, with higher FDG uptake in the inner circular layer compared to the surrounding outer layer. These findings were further supported by the increased expression of GLUT1 in synthetic VSMCs of neointima region. Although further studies are warranted to clarify the mechanisms underlying this stratified distribution, we hypothesize that the synthetic activity of VSMCs may be greater in the early phase of neointimal hyperplasia than in the later phases.

F-18 FDG PET is a potentially useful tool for evaluating the activity of synthetic VSMCs in a wide range of vascular remodeling diseases. In atherosclerosis, synthetic VSMCs induce neointimal hyperplasia, narrow the lumen, and provide substrates for lipoprotein retention, thereby accelerating the progression of atherosclerosis^19^. In addition to synthetic VSMCs, inflammatory responses also play a key role in vascular wall damage in atherosclerosis^20^. In particular, macrophages appear to significantly contribute to foam cell formation and plaque rupture^20^. While neointimal hyperplasia is also formed in in-stent restenosis and transplant vasculopathy, inflammatory responses appear to play only a minor role in these vasculopathies^19^. Recently, Kim *et al*.^21^ reported that ^68^Ga-labeled NOTA-neomannosylated human serum albumin (MSA) could target macrophages in atherosclerosis. Using a combination of FDG and MSA, the molecular basis of synthetic VSMC and macrophage activities in atherosclerotic lesions could be easily obtained.

## Conclusion

The activity of synthetic VSMCs was successfully visualized by F-18 FDG PET. Therefore, F-18 FDG PET is a promising noninvasive imaging modality for evaluating synthetic VSMCs, especially in vascular remodeling disorders.

## Materials and Methods

### Animals

Eight male Sprague-Dawley (SD) rats (7 weeks old, 200 g body weight) were purchased from Orient-Bio (Seongnam, Korea). 4 rats were enrolled in atherosclerotic group and 4 rats were in normal control group. All rats were maintained under a 12-h/12-h day/night cycle with *ad libitum* water and meals. All experimental protocols and procedures were approved by the Ethics Committee and the Institutional Animal Care and Use Committee of Korea University College of Medicine (Approval No. KOREA-2016-0041). All experiments were performed in accordance with the approved guidelines and regulations.

### Induction of atherosclerosis

After a 1-week adaptation period, right partial carotid artery ligation was performed as previously described^9^. Briefly, anesthesia was induced with 3.5% isoflurane with a 2:1 N_2_O/O_2_ mixture in a vented anesthesia chamber and then maintained by the administration of 2 to 2.5% isoflurane with a 2:1 N_2_O/O_2_ mixture through a nasal cone. The right external carotid artery, right internal carotid artery, and right occipital artery were ligated with a 6-0 silk suture. After ligation, vitamin D3 (6 × 10^5^ IU/kg) was intraperitoneally injected for 2 consecutive days^22^, with the exception of the normal control group. Rebsamen *et al*.^23^ reported that vitamin D3 facilitates VSMC migration in the SD rat aorta. The atherosclerotic group was fed daily with a commercially available atherogenic diet (D12336, Research Diets, NJ, USA) for 4 weeks. The normal group underwent a sham operation without ligation of carotid arteries or vitamin D3 injections. Normal rats were fed a diet of normal chow for 4 weeks.

### ^18^F-FDG PET imaging and analysis

Images were acquired using a small animal PET/CT (computed tomography) scanner (eXplore Vista DR PET/CT, GE Healthcare, Milwaukee, WI, USA). Rats were not fasted before examination and were anesthetized with 2% isoflurane at 1 L/min oxygen flow during the PET/CT scan. Rats were placed in the prone position under the scanner. PET image acquisition started 40 min after the injection of 37 MBq/0.2 ml of F-18 FDG via the tail vein. Static PET scans were acquired for 20 min in a single bed position covering the carotid artery region. The axial field of view (FOV) of the PET scanner was 48 mm in length. CT scanning (40 kV, 250 µA) was initiated after PET scanning of the same area. Images were reconstructed using Fourier rebinning and the ordered subsets expectation maximization (OSEM) algorithm with decay, attenuation, random, and normalization corrections. The voxel size was 0.3875 × 0.3875 mm; axial slice thickness was 0.775 mm. Maximum intensity projection (MIP) images, axial views, sagittal views, and fusion images were processed after reconstruction. Following image reconstruction, image analysis was performed with A Medical Image Data Examiner Software (AMIDE, version 1.0.4)^24^. On each image, regions of interest (ROI) were drawn over the carotid artery region and the standardized uptake values (SUVs) were measured. The SUV was calculated as *activity concentration* (*ROI; MBq/ml*)*/injected dose* (*MBq*)*/total body weight* (*g*).

### Autoradiography

Immediately after completion of PET/CT scanning, the carotid arteries were harvested, fixed in 4% paraformaldehyde (PFA), and mounted on glass slides. The prepared tissues were exposed to imaging plates and images were acquired using a BAS-200 system (FLA-2000, FUJIFILM, Tokyo, Japan).

### Immunohistochemistry

Harvested carotid arteries were fixed with 4% PFA and preserved in 30% sucrose solution. Tissues were embedded in Optimal Cutting Temperature (OCT) compound (Scigen Scientific, Gardena, CA, USA). Axial sections of 4-μm thickness were cut using a cryostat microtome (Leica CM 3050 S, Leica Microsystems, Wetzlar, Germany). The molecular profiles of the carotid artery lesions were investigated by immunofluorescence. To this end, sections were blocked in 5% goat serum in phosphate-buffered saline (PBS) with 0.1% Triton X-100 for 60 min and incubated at 4 °C with the following antibodies: anti-α-SMA (1:200 dilution, ab7817 or ab5694, Abcam, Cambridge, MA, USA), anti-SM-MHC (1:200 dilution, ab683, Abcam, Cambridge, MA, USA), anti-CD68 (1:200 dilution, MCA341R, Serotec, Oxford, UK), anti-myeloperoxidase (1:800 dilution, A0398, DAKO, Glostrup, Denmark), anti-cyclophilin A (1:100 dilution, ab41684, Abcam, Cambridgem MA, USA), anti-MMP-9 (1:200 dilution, AB19016, Merck Millipore, Billerica, MA, USA), anti-collagen type III (1:200 dilution, ab7778, Abcam, Cambridge, MA, USA), and anti-GLUT1 (1:200 dilution, ab652, Abcam, Cambridge, MA, USA). Alexa Fluor 555-conjugated goat anti-mouse IgG (1:400 dilution, A21424, Invitrogen, Carlsbad, CA, USA) and Alexa Fluor 488-conjugated goat anti-rabbit IgG (1:400 dilution, A11034, Invitrogen, Carlsbad, CA, USA) were used as secondary antibodies. Cell nuclei were counterstained with DAPI. Histopathologic evaluation was performed by staining with hematoxylin and eosin. All images were acquired on a confocal microscope (LSM800, Carl Zeiss, Oberkochen, Germany) or an upright light microscope (BX51, Olympus, Tokyo, Japan).

### Western blotting

Harvested carotid arteries were homogenized in RIPA buffer (GeneDepot, Barker, TX, USA) and centrifuged at 13,000 rpm for 15 min at 4 °C. The protein concentration of supernatant was determined using bicinchoninic acid reagent (Thermo Fisher, Waltham, MA, USA). Proteins were separated using an 8% sodium dodecyl sulfate polyacrylamide gel electrophoresis gel and transferred to a polyvinyldine fluoride membrane (PVDF, Merck Millipore, Billerica, MA, USA). The membranes were blocked for 1 h with 5% skim milk in TBS-T (20 mM Tris-HCl (pH 7.6) containing 0.8% NaCl and 0.1% Tween 20). Then, the membranes were incubated with antibodies against GLUT1 (1:500 dilution, ab15309, Abcam, Cambridge, MA, USA) and glyceraldehyde 3-phosphate dehydrogenase (GAPDH) (1:2000 dilution, MAB374, Merck Millipore, Billerica, MA, USA). After washing with TBS-T, it was incubated with goat anti-rabbit or anti-mouse horseradish peroxidase conjugated secondary antibodies (1:30000 dilution, A16110 or 31430, Thermo Fisher, Waltham, MA, USA). To obtain protein bands, PVDF membranes were detected with X-ray film.

### Statistical analysis

The Mann-Whitney *U* test was used as a statistical method using SPSS software version 17.0 (SPSS Inc, Chicago, IL, USA). A *p*-value < 0.05 was defined as statistically significant.

## Electronic supplementary material

Supplementary information
