# Supplementary material for: Visualization of Synthetic Vascular Smooth Muscle Cells in Atherosclerotic Carotid Rat Arteries by F-18 FDG PET
Source: Sci Rep. 2017 Aug 1;7:6989. doi: 10.1038/s41598-017-07073-3 (PMC5539104; doi:10.1038/s41598-017-07073-3)
Supplement: Supplementary file 1 — Supplementary information [file 41598_2017_7073_MOESM1_ESM.pdf]

Supplementary Information

Visualization of Synthetic Vascular Smooth Muscle Cells in Atherosclerotic Carotid Rat Arteries by F-18 FDG PET

Kisoo Pahk[1, 2], Chanmin Joung[1], Se-Mi Jung[1], Hwa Young Song[1], Ji Yong Park[3, 4], Jung Woo Byun[3], Yun-Sang Lee[3, 5], Jin Chul Paeng[6], Chunsook Kim[7], Sungeun Kim[2**], and Won-Ki Kim[1*]

[1]*Department of Neuroscience, Korea University College of Medicine, Seoul, Korea;*

[2]*Department of Nuclear Medicine, Korea University Anam Hospital, Seoul, Korea;*

[3]*Department of Nuclear Medicine, Seoul National University College of Medicine, Seoul, Korea;*

[4]*Department of Biomedical Sciences, Seoul National University Graduate School, Seoul, Korea;*

[5]*Department of Molecular Medicine and Biopharmaceutical Sciences, Graduate School of Convergence Science and Technology, and College of Medicine, Seoul National University, Seoul, Korea;*

[6]*Department of Nuclear Medicine, Seoul National University Hospital, Seoul, Korea;*

[7]*Department of Nursing, Kyungdong University, Wonju, Korea*

***Corresponding Author:**

Won-Ki Kim, Ph.D.

Professor, Department of Neuroscience

Korea University College of Medicine

126-1, Anam-Dong 5-Ga, Seongbuk-Gu, Seoul 136-705, Korea

Tel: 82-2-2286-1095, Fax: 82-2-953-6095, E-mail: wonki@korea.ac.kr

**** Co-corresponding Author:**

Sungeun Kim, M.D., Ph.D.

Professor, Department of Nuclear Medicine

Korea University Anam Hospital

126-1, Anam-Dong 5-Ga, Seongbuk-Gu, Seoul 136-705, Korea

Tel: 82-2-920-5540, Fax: 82-2-921-2971, E-mail: seiong@korea.ac.kr

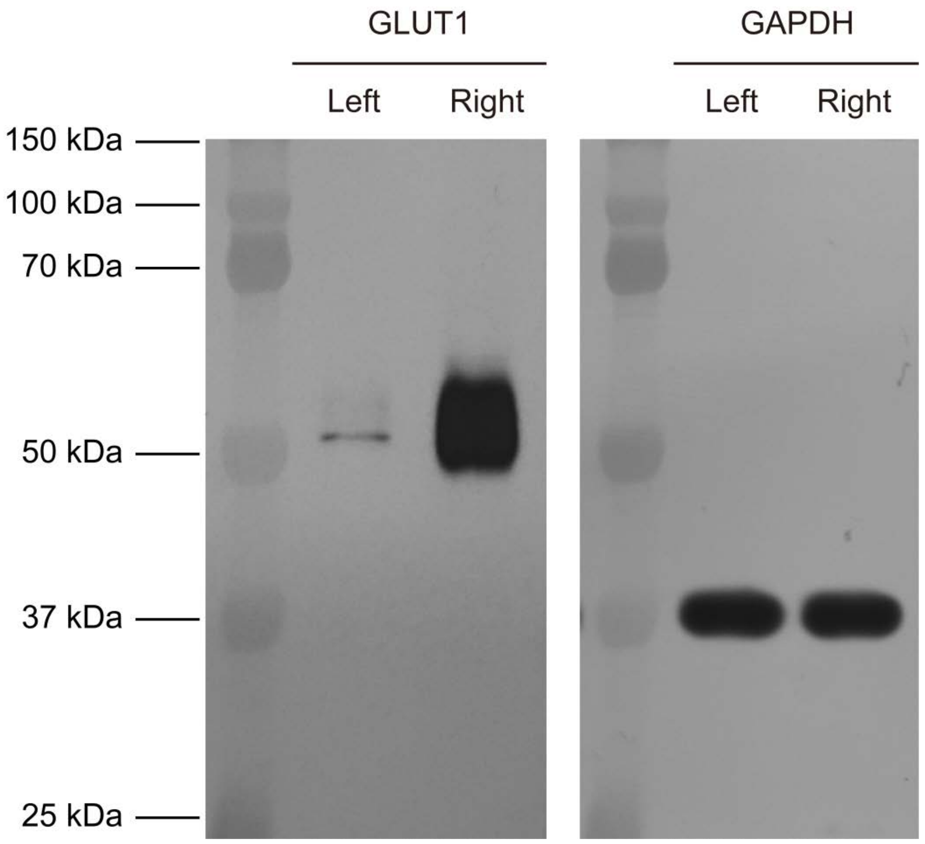

Supplementary Figure 1. Immunoblot images of GLUT1 and GAPDH expressions. The cropped images are shown in Figure 6B.
